# Supplementary material for: PICH acts as a force-dependent nucleosome remodeler
Source: Nat Commun. 2022 Nov 25;13:7277. doi: 10.1038/s41467-022-35040-8 (PMC9700735; doi:10.1038/s41467-022-35040-8)
Supplement: Supplementary file 1 — Supplementary Information [file 41467_2022_35040_MOESM1_ESM.pdf]

Supplementary Information

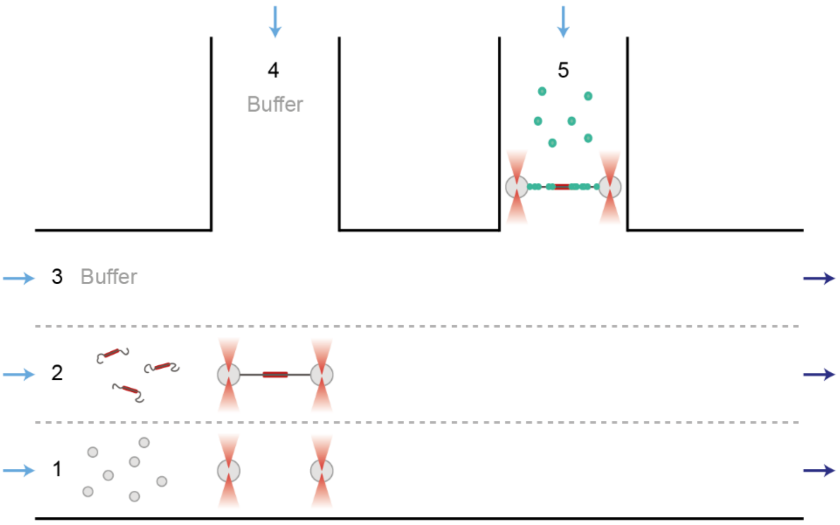

Supplementary Fig. 1: Schematic representation of the 5-channel microfluidics flow cell used for single-molecule experiments. Channel 1 contained streptavidin-coated polystyrene beads (grey) that can be optically-trapped. Channel 2 contained biotinylated nucleosome-array constructs (red; with or without fluorescent labels on the histones). Channel 3 and 4 contained only measurement buffer with 2 mM ATP (or AMP-PNP). Channel 5 contained measurement buffer with 25 nM (or 100 nM) PICH-eGFP (green) and 2 mM ATP (or AMP-PNP). To perform constant-force measurements, two beads were first optically-trapped in channel 1. These trapped beads were then moved to channel 2, where a nucleosome-array construct was tethered between the beads. For the constant-force measurements, this construct was then moved, via channel 3, to channel 5. Here, the constant-force measurement is either directly performed, or the construct is only coated with PICH, and subsequently moved to channel 4 to prevent additional PICH from binding. The direction of flow is depicted by arrows: light blue for the inlet flow and purple for the outlet flow. Flow is turned on to catch beads and tether the nucleosome-array construct, and turned off throughout the rest of the experiment.

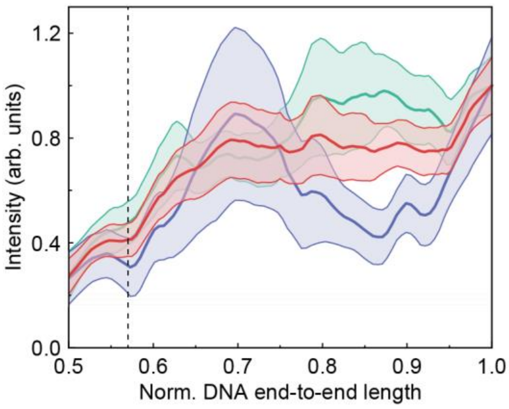

Supplementary Fig. 2: Lack of PICH binding to nucleosome area at ~1 pN is independent of the presence of Anti-H3-Alexa647 antibodies. Mean intensity profiles of PICH-eGFP along nucleosome-array constructs that were used for the bottom panel of Fig. 1b (i) (red). These PICH-eGFP intensity profiles were further divided by the absence (green) or presence (purple) of Anti-H3-Alexa647 antibodies on the corresponding nucleosome-array constructs. The dashed black line indicates the area of nucleosome positioning sequences on the array. N=10 and N=4 for non-labelled (green) and labelled (purple) nucleosome-array constructs. Shaded areas represent the ±SEM. Source data are provided as a Source Data File.

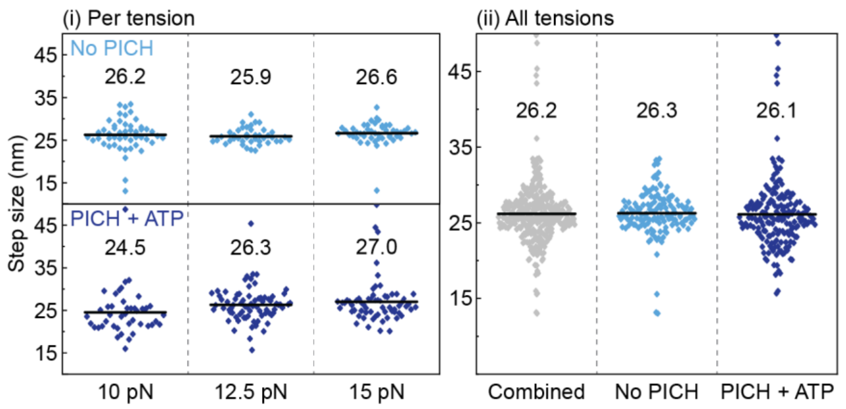

Supplementary Fig. 3: Step sizes of DNA end-to-end lengthening events corresponding to inner-turn unwrapping of nucleosomes. (i) Scatter plots showing the measured individual step sizes (see Methods) in the absence (light blue) or presence (purple) of PICH and ATP, at 10, 12.5 and 15 pN. For 10, 12.5 and 15 pN, *N*=50, 41 and 48 in the absence of PICH and ATP and *N*=45, 74 and 67, in the presence of PICH and ATP. (ii) All step sizes combined (grey, *N*=355) and categorized by the absence (light blue, *N*=140) or presence (purple, *N*=215) of PICH and ATP. Mean values are indicated by both horizontal lines and corresponding numbers. Source data are provided as a Source Data File.

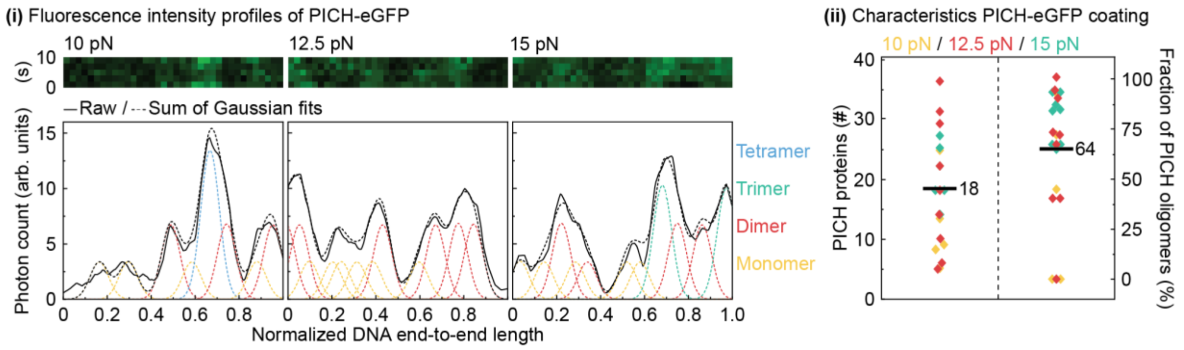

Supplementary Fig. 4: The majority of PICH bound to the nucleosome-array construct is present as apparent oligomers. (i) Representative kymographs (*upper*) and plot profiles (*lower*, black solid lines) of PICH-eGFP during the first ten seconds of incubation in 25 nM PICH and 2 mM ATP, at 10 pN (*left*), 12.5 pN (*middle*) and 15 pN (*right*). The width of the kymographs corresponds to ~2.9 µm. Peaks of the plot profiles were fitted with Gaussians of the point spread functions of either single PICH-eGFP proteins (i.e., monomers, yellow dashed lines) or apparent PICH-eGFP oligomers (e.g., dimers, red dashed lines) (see Methods). The legend shows the colours corresponding to the different oligomeric states. Dashed black line represents the cumulative curve of all Gaussians fits. (ii) Scatter plots showing the mean number of PICH proteins per nucleosome-array construct and the fraction of PICH proteins present as apparent oligomers at 10 pN (yellow), 12.5 pN (red) and 15 pN (green) (*N*=5, 9 and 8 constructs, respectively). Overall mean values are indicated by black horizontal lines and corresponding numbers. Source data are provided as a Source Data File.

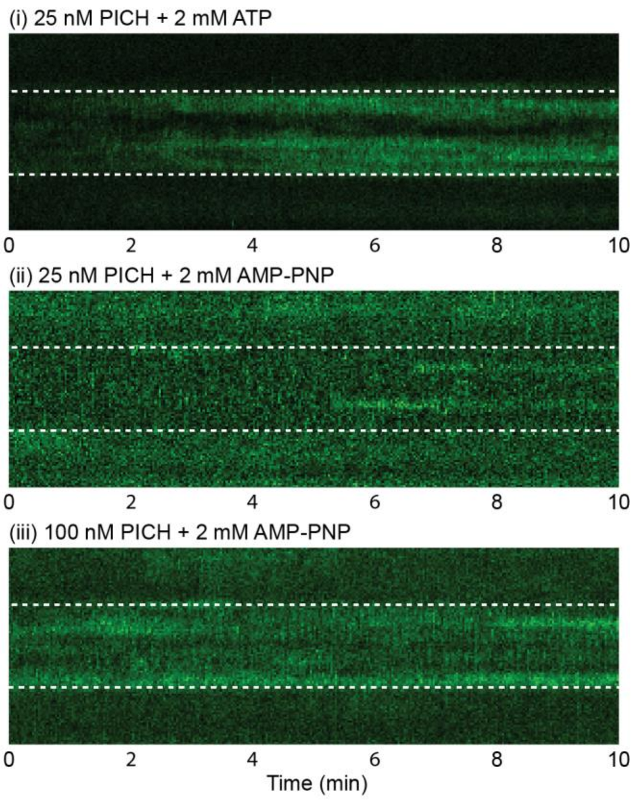

Supplementary Fig. 5: Using AMP-PNP in place of ATP affects the DNA binding properties of PICH. Representative kymographs showing the interaction of PICH-eGFP with a nucleosome-array construct over time (at 10 pN) under the following conditions: (i) 25 nM PICH-eGFP + 2 mM ATP; (ii) 25 nM PICH-eGFP + 2 mM AMP-PNP; and (iii) 100 nM PICH-eGFP + 2 mM AMP-PNP. Each dashed white box indicates the area of the nucleosome-array construct, which corresponds to 3 µm. Source data are provided as a Source Data File.

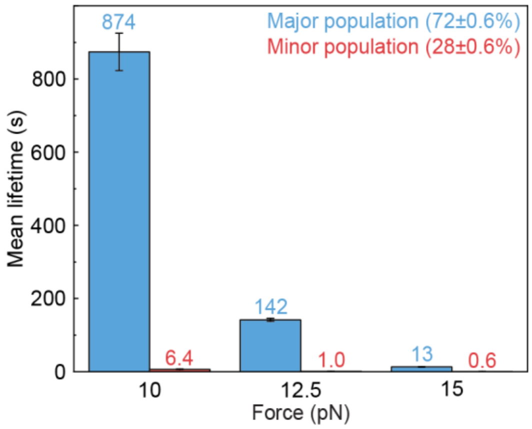

Supplementary Fig. 6: Bar chart the showing mean lifetimes of the major (light blue) and minor (red) population of ‘nucleosomes’ as determined by the bi-exponential fits shown in Fig. 3b. Data are presented as mean lifetimes ± standard error of the fits. N=50, 41, 48 for 10, 12.5 and 15 pN, respectively. Source data are provided as a Source Data File.

Supplementary Table 1: Settings used for fluorescence imaging

Experiment	488 nm laser (mW)	638 nm laser (mW)	Pixel dwell time (ms)	Inter-frame wait time (s)
Snapshots of PICH-eGFP and Anti-H3-Alexa647	2.0	0.50	0.5	-
Kymographs of PICH-eGFP and Anti-H3-Alexa647	3.8	0.02	1.0	2.0
Kymographs of PICH-eGFP and Atto-647N	3.8	0.02	0.2	1.0
Kymographs of PICH-eGFP	2.0	-	0.5	2.0
