# Supplementary material for: PICH acts as a force-dependent nucleosome remodeler
Source: Nat Commun. 2022 Nov 25;13:7277. doi: 10.1038/s41467-022-35040-8 (PMC9700735; doi:10.1038/s41467-022-35040-8)
Supplement: Supplementary file 3 — Reporting Summary [file 41467_2022_35040_MOESM3_ESM.pdf]

nature portfolio

Corresponding author(s): Erwin J.G. Peterman
Gijs J.L. Wuite

Last updated by author(s): Nov 10, 2022

Reporting Summary

Nature Portfolio wishes to improve the reproducibility of the work that we publish. This form provides structure for consistency and transparency in reporting. For further information on Nature Portfolio policies, see our Editorial Policies and the Editorial Policy Checklist.

Statistics

For all statistical analyses, confirm that the following items are present in the figure legend, table legend, main text, or Methods section.

n/a	Confirmed	
☐	☒	The exact sample size (n) for each experimental group/condition, given as a discrete number and unit of measurement
☒	☐	A statement on whether measurements were taken from distinct samples or whether the same sample was measured repeatedly
☒	☐	The statistical test(s) used AND whether they are one- or two-sided<br>*Only common tests should be described solely by name; describe more complex techniques in the Methods section.*
☒	☐	A description of all covariates tested
☐	☒	A description of any assumptions or corrections, such as tests of normality and adjustment for multiple comparisons
☐	☒	A full description of the statistical parameters including central tendency (e.g. means) or other basic estimates (e.g. regression coefficient) AND variation (e.g. standard deviation) or associated estimates of uncertainty (e.g. confidence intervals)
☒	☐	For null hypothesis testing, the test statistic (e.g. F, t, r) with confidence intervals, effect sizes, degrees of freedom and P value noted<br>*Give P values as exact values whenever suitable.*
☒	☐	For Bayesian analysis, information on the choice of priors and Markov chain Monte Carlo settings
☒	☐	For hierarchical and complex designs, identification of the appropriate level for tests and full reporting of outcomes
☒	☐	Estimates of effect sizes (e.g. Cohen's d, Pearson's r), indicating how they were calculated

Our web collection on statistics for biologists contains articles on many of the points above.

Software and code

Policy information about availability of computer code

Data collection	We used custom written Labview software (LV 2011, as used previously in Heller et al., Nat. Methods 10, 910-916 (2010)) to record Force-Distance (FD) and Distance-Time (DT) curves and confocal fluorescence data.
Data analysis	We used a custom written Labview program (LV 2011, see above) to extract FD and DT curves, a custom written MATLAB program (ML 2016b) to determine nucleosome inner-turn unwrapping events, Python 3.7 to perform logistic regression analysis, ImageJ v1.53c to analyze fluorescence data, and OriginPro 2019 to analyze both FD/ DT curves and fluorescence data.

For manuscripts utilizing custom algorithms or software that are central to the research but not yet described in published literature, software must be made available to editors and reviewers. We strongly encourage code deposition in a community repository (e.g. GitHub). See the Nature Portfolio guidelines for submitting code & software for further information.

Data

Policy information about availability of data

All manuscripts must include a data availability statement. This statement should provide the following information, where applicable:

- Accession codes, unique identifiers, or web links for publicly available datasets
- A description of any restrictions on data availability
- For clinical datasets or third party data, please ensure that the statement adheres to our policy

Source data are provided with this paper. Additionally, all data generated in this study have been deposited in the Dataverse repository at https://doi.org/10.34894/RDQLB5.

Human research participants

Policy information about studies involving human research participants and Sex and Gender in Research.

Reporting on sex and gender	Not applicable
Population characteristics	Not applicable
Recruitment	Not applicable
Ethics oversight	Not applicable

Note that full information on the approval of the study protocol must also be provided in the manuscript.

Field-specific reporting

Please select the one below that is the best fit for your research. If you are not sure, read the appropriate sections before making your selection.

- [x] Life sciences
- [ ] Behavioural & social sciences
- [ ] Ecological, evolutionary & environmental sciences

For a reference copy of the document with all sections, see nature.com/documents/nr-reporting-summary-flat.pdf

Life sciences study design

All studies must disclose on these points even when the disclosure is negative.

Sample size	No calculations were performed to predetermine sample sizes. All observations related to fluorescence are based on a minimum of 4 nucleosome array constructs per condition. All observations on nucleosome unwrapping events are based on a minimum of 20 nucleosomes from at least 4 nucleosome array constructs per condition.
Data exclusions	For quantifications of nucleosome unwrapping, nucleosome array constructs were excluded if they contained fewer than 2 nucleosomes. For fluoresence analysis, nucleosome array constructs were excluded if no histones were labelled.
Replication	All data were collected from a minimum of 4 unique nucleosome array constructs per condition. All attempts at replication were successful and gave similar results.
Randomization	This does not apply for this study, since data in single-molecule studies are inherently randomized.
Blinding	In our single-molecule experimental set-up, blinding during data collection is inherently not possible. No blinding was applied during data analysis. However, standardized procedures for data collection and analysis were used to prevent bias. Additionally, both first authors analyzed most of the obtained data in parallel and found no notable differences.

Reporting for specific materials, systems and methods

We require information from authors about some types of materials, experimental systems and methods used in many studies. Here, indicate whether each material, system or method listed is relevant to your study. If you are not sure if a list item applies to your research, read the appropriate section before selecting a response.

Materials & experimental systems

n/a	Involved in the study
	☒ Antibodies
	☒ Eukaryotic cell lines
☒	Palaeontology and archaeology
☒	Animals and other organisms
☒	Clinical data
☒	Dual use research of concern

Methods

n/a	Involved in the study
☒	ChIP-seq
☒	Flow cytometry
☒	MRI-based neuroimaging

Antibodies

Antibodies used	Anti-H3-Alexa647: Thermo Fisher Scientific, Catalog #MA7-02023-A647, Clone 17H2L9, Lot #: VC294388.
Validation	This commercially available antibody was validated by the manufacturer, Thermo Fisher Scientific, for use in ICC and IF in human cells.

Eukaryotic cell lines

Policy information about cell lines and Sex and Gender in Research

Cell line source(s)	Sf21 insect cells were used to express recombinant human PICH-eGFP.
Authentication	Cell line was not authenticated, as this is generally not needed for highly identifiable insect cells.
Mycoplasma contamination	Cell line was not tested for mycoplasma contamination.
Commonly misidentified lines (See ICLAC register)	Not applicable.
